# COVID-19: the trends of conspiracy theories vs facts

**DOI:** 10.11604/pamj.supp.2020.35.147.25536

**Published:** 2020-08-17

**Authors:** Favour Obianujunwam Bernard, Jethro Aaron Akaito, Isaac Joseph, Kenneth Bitrus David

**Affiliations:** 1 Faculty of Pharmacy, University of Nigeria Nsukka, Enugu, Nigeria,; 2 Department of Chemistry, Kaduna State University, Kaduna, Nigeria,; 3 Faculty of Pharmaceutical Sciences, Kaduna State University, Kaduna, Nigeria

**Keywords:** COVID-19, SARS-CoV-2, conspiracy theories, facts

## Abstract

Coronavirus disease (COVID-19) caused by SARS-CoV-2-a new single-stranded RNA virus with respiratory system proclivity and epithelial cell- is a novel infectious disease that originated in Wuhan, China in December, 2019 and has spread to many countries with the total number of confirmed cases put at 20,259,579 cases as of 12^th^ August, 2020. It is transmitted from human-to-human via droplets. When an infected person coughs or sneezes, these droplets find their way into the mouth or nostrils of another person that is within a close range. Alternatively it can be contracted by touching infected hard surfaces and using the same hands to touch the mouth, nose and eye(s). COVID-19 has been declared a global pandemic by the World Health Organization (WHO) on 11th March, 2020. There is currently no therapeutic substance accepted as a panacea for the prophylaxis of this infectious disease. As a result of this back drop, many nations have instituted fourteen (14) days quarantine for suspected cases, social distancing and border closure in an attempt to curb the spread of COVID-19. There has been several conspirary theories that emanated since the disease was declared a pandemic. This paper provides useful information to serve as reference to those who seek proper understanding of COVID-19 and its deleterious effects in the body, by distiguishing between the factsand the conspiracy theoriesof coronavirus disease.

## Commentary

**History of COVID-19:** COVID-19 which stands for Corona virus Disease 2019, is an infectious disease caused by a newly discovered coronavirus strain-SARS-CoV-2 [1]. The novel corona virus was discovered in China, linked to a wholesale market in Wuhan. The mortality rate seems to be higher in children and older people with underlying medical problems such as cardiovascular diseases, diabetes, chronic respiratory diseases and cancer. Most of the younger people infected with SARS-CoV-2 will experience mild to moderate respiratory illness and recover without special treatment. Some deaths were however recorded in middle-aged patients. Some people are asymptomatic carriers; they can harbor this virus without showing any significant symptoms. The virus spreads through droplets from the respiratory tract, when an infected person coughs or sneezes, therefore it is recommended that everyone wears a face mask, maintain a level of distance from other people, and also adhere to other precautionary measures so as to minimize the spread of the virus. Conspiracy has been built on the idea that corona virus is not new, a suspicion that has manifested transversely across countries. Origin based theories defend the artificial nature of the virus which spread either accidentally or intentionally. The accident-centered thread blames without evidence, the virus spread on the carelessness of the actors involved, who conducted convert activities (e.g. a bioweapon program went wrong). The exploitation of resources and consumers are also pillowed as some suggest a relation of causality between the epidemic and intensive breeding.

Multiple Russian sources of deformation subsequently echoed in other countries, emphasizing the bacteriological war intent of the pandemic with assumptions that it originated in the United States of America or China. Accordingly, the virus was created in a lab and was released voluntarily in order to achieve geopolitical (e.g. a made up story circulated in Italy that an American veteran was paid to spread the virus to Europe) or economic gains( e.g. A French research center was accused of creating the virus in 2004 in order to sell its patented vaccine). The disease was initially reported to WHO on December 31^st^, 2019. On January 30^th^2020, WHO declared the COVID-19 outbreak a global health emergency, and on March 11^th^2020, it was declared a global pandemic by WHO [2]. In late March and early April, president Trump of the United States of America repeatedly proclaimed that hydroxychloroquine could prevent or treat COVID-19 [3]. Within few days, the number of prescription for the drug increased even though there were no substantial evidences as to its efficacy in the treatment of the viral disease [3].

**Trends of conspiracy theories vs facts:** since the emergence of the global lockdown caused by COVID-19, there has been a lot of controversy headed by groups of people about the global health issue which gainsay the underlined facts by the governing bodies and health practitioners. From the inception of this global pandemic, there has always been a contention of who knows better amongst researchers, doctors and the citizens. It is as if we are living in the same building but split into two parts. Many people still doubt the existence of the COVID-19 virus, to some it is just a game of politics. Looking at the coronavirus pandemic, conspiracy theories range from entirely fabricated content to the instrumentation of tentative or speculative findings to which absolute certainty is attributed in what resembles a game of Chinese whispers [4].

**Political manipulation or how to use secret agenda:** conspiracy theories advocate the existence of secret plots which the powerful elites hide from the public's eye in a bid to politically manipulate the people. Many forms of conspiracies have been ongoing in several countries owing to how the government has used the COVID-19 as a tool for getting funds from other nations. Thus there has been trends of speculations that the number of confirmed cases are been inflated. In Nigeria, for instance, there are reports that some patients who visited the hospital gave information of how a mere malaria case was recorded as a COVID-19 case. In Nigeria, the citizens are still puzzled on how the Nigeria Centre for Disease Control (NCDC) treats the COVID-19 patients since WHO has cancelled the use of hydroxychloroquine. Many Nigerians still doubt the NCDC testing capacity. In a viral video released via the social media, a lady was seen dancing continuously to the chorus “*Do you know somebody who knows somebody, who has corona*” Which literally calls corona virus especially in Nigeria a hoax. Majority of Nigerians are apoplectic noting that even at such a high number of COVID-19 cases, none of them have ever seen a victim of the disease, the only ones they have seen in a video is just a group of NCDC officials engaging in mortal combat with their accused victims. Also there have been claims in Nigeria at the beginning of the lockdown due to the pandemic that political activities were still holding while schools, markets, worship centers were closed. In recent time, in the metropolis of Kaduna, Nigeria, all the shops at Kaduna Central Market have been closed in order to reduce large gathering of people, but sadly, all the shop owners resorted to the display of their products on the street, outside the main market, consequently leading to congestion of people thereby making the initial intent for the market closure futile. A further step in the narrative conspiracy proposes the reluctance of the government to communicate the real situation. Accordingly, the goal is to push an undemocratic and unethical agenda that would cause public upheavals if known.

**Population cleansing is a widespread theory that emerged in different terms:** examples of conspiracy theories in this class include claims that China created the virus to solve the overpopulation problem, whereas in Europe the virus was to eliminate the elderly. In Nigeria, the conspiracy theory is that the virus has come to eliminate the corrupt political leaders of the country. It is also feared that the pandemic will be used as an excuse to imposed meddles mass vaccination, whose real purpose is to implement Orwellian mechanism of social control. In addition other conspiracy theories suggest that the lockdown is an excuse to force people in their homes, while authoritarian measures are been implemented. A common goal is to show the collusion of political and economic powers for evil intent.

**Evergreen Conspiracies Step into the shoes of COVID-19:** besides the extensively explored deep state allegations, other conspiracy theories that do not entirely fit the previous narratives include: 1) The prophetic origin of SARS-CoV-2 foreseen by Nostradamus, Bill gates or the Simpsons. 2) A revival of anti-sanitism accusing Jewish communities of speeding the virus for the purpose of poisoning the gentiles and sacrificing them as part of religious rituals. 3) Finally, the 5G conspiracy has also become a narrative of its own primed in Europe and beyond. Popular claims are that SARS-CoV-2 is transmitted through 5G antennas and that the 5G technology has a negative health impact and thus make individuals vulnerable to the virus.

**Cure and medical treatment conspiracy theories:** a number of conspiracy theories confuse the long-standing existence of COVID-19, therefore claiming that the cure already existed and in line with the theory of power, the elite had refused to share it with the whole population. To illustrate this, in France some group of Facebook users met the news of the speedy recovery of Prince Charles despite his old age with suspicion. In Nigeria there has been many conspiracies about the existence of the disease owing that many of the mortality cases occurs in the elderly age and there has been trend that people in the age died of merely terminal disease that is co infection rather than due to the virus. According to a research which was carried out by the Solidarity Trials International Steering committee which was later accepted and approved by the World Health Organization; WHO, showed that the drug hydroxychloroquine and some anti-viral drugs Like lopinavir/ritonavir produce little or no reduction in the mortality of hospitalized COVID-19 patients when compared to standard of care [5]. WHO accepted their recommendation and discontinued the treatment of COVID-19 patients with hydroxychloroquine and lopinavir/ritonavir. In a recent video released by Dr. Stella Immanuel, she made an unsubstantial claim that hydroxychloroquine is a cure for COVID-19; the disease caused by SARS-CoV-2. She also claimed that she has cured about 350 corona virus patients noting that a mixture of hydroxychloroquine, azithromycin and zinc sulphate is a permanent cure for their virus. She added also that neither face mask nor lockdown are necessary to fight the pandemic [6]. She accused other doctors, health practitioners and political heads of state of concealing the truth from the people. Her video was also retweeted by Trump to show his support. The video was deleted by all social media platforms and termed to be violating COVID-19 rules and misleading. Remember that Trump had also frozen US government funding to WHO due to conspiracy on how it handles COVID-19 pandemic. Also recall that Madagascar purported a homemade herbal cure for corona virus, which the president, Andry Rajoelina applauded and also added that he was paid millions of dollars by WHO to discontinue the production of the drug, but as of 9^th^August, the number of confirmed COVID-19 patients has reached to13,086 with about 2,122 active cases and 148 deaths [7,8]. One will question if the herbal drug does not work again?

## Conclusion

Many conspiracies have evolved due to this novel coronavirus disease, some having some facts that could be true. Amongst all these conspiracy, nevertheless, Corona virus disease exists and is a pandemic that may stand the test of time for a while before its elimination. The truth and the fact still remains that as for now, there is no approved permanent medication or prescription that can efficiently combat the corona virus and the best treatment one could offer to oneself is keeping the precautionary measures outlined by WHO and the government, which includes; regular washing of hands, use of hand sanitizers, maintaining physical distance up to one-feet apart. Most importantly, we should be wise enough to stop the spread of fake news concerning COVID-19 and linking everything to religious misconception, Nonetheless, Vaccines have been proposed and not yet proved to be efficacious. Note also “*prevention is better than cure and a healthy man is a wealthy man*”.
